# Efficacy of Intravenous Nefopam, Dexmedetomidine, and Meperidine in Preventing Postspinal Anesthesia Shivering in Adult Patients Undergoing Lower Abdominal and Lower Limb Surgeries: A Double‐Blind Comparative Study

**DOI:** 10.1155/anrp/4549345

**Published:** 2025-12-03

**Authors:** Emad M. Abdelhafez, Wael El-Siory, Dina Turki, Amany A. Eissa

**Affiliations:** ^1^ Anesthesia, Surgical Intensive Care and Pain Management Department, Faculty of Medicine, Cairo University, Cairo, Egypt, cu.edu.eg

**Keywords:** dexmedetomidine, meperidine, nefopam, shivering, spinal anesthesia

## Abstract

**Background:**

A common and distressing consequence that may arise after spinal anesthesia (SA) is shivering. Research focuses on the role of intravenous (IV) nefopam, dexmedetomidine (DEX), and meperidine in averting shivering episodes in adult individuals receiving SA for surgical interventions on the lower abdomen or limbs.

**Methods:**

This prospective, randomized, controlled, double‐blind trial involved 210 patients, aged 21–60 years, of both sexes, scheduled for abdominal or limb surgeries under SA. Patients were equally randomized into three groups: Groups N, D, and P received IV nefopam (0.2 mg/kg), DEX (0.5 μg/kg), and pethidine (0.5 mg/kg), respectively. The medications were infused over 20 min.

**Results:**

Heart rate and mean arterial pressure were significantly increased in Groups N and P as opposed to Group D (*p* < 0.05), with no considerable difference between Groups N and P. Oxygen saturation and core temperature remained similar across all groups at all measurement points. The incidence of shivering was significantly lower in Group N (6 [8.57%]) as opposed to Group P (19 [27.14%]) and Group D (39 [55.71%]) (*p* < 0.001). Shivering onset, grade, duration, frequency of rescue drug administration, and total rescue drug dosage were comparable among groups. However, sedation levels were significantly lower in Groups N (1 [1–2]) and P (2 [1–2]) as opposed to Group D (2 [1–2]) (*p* < 0.001 and *p* = 0.009), with no significant difference between Groups N and P (*p* = 0.06).

**Conclusions:**

IV nefopam was more effective than DEX and pethidine in preventing shivering under SA, with fewer hemodynamic and sedative side effects.

**Trial Registration:**

ClinicalTrials.gov identifier: NCT06627816

## 1. Introduction

Shivering after spinal anesthesia (SA) is a common and annoying side effect of regional anesthesia. Its incidence ranges from 36% to 65%, depending on factors such as ambient temperature and patient characteristics [1]. Shivering presents significant discomfort for patients and poses a clinical challenge for anesthesiologists, as it can compromise the reliability of intraoperative monitoring. Furthermore, it initiates a cascade of physiological alterations, including increased metabolic rate and oxygen consumption, which may result in arterial hypoxemia [2].

The pathophysiology of postspinal shivering is primarily attributed to thermoregulatory dysfunction caused by regional anesthesia‐induced vasodilation and inhibition of central thermoregulation [3].

SA impairs afferent thermal input and reduces the shivering threshold, triggering heat‐conserving responses such as vasoconstriction and shivering via the hypothalamic median preoptic nucleus [3]. Consequently, effective management of shivering is critical not only for patient comfort but also for preventing adverse events (AEs) and optimizing perioperative metabolic stability [4].

Painkillers, serotonergic medications, Alpha 2 agonists, and NMDA antagonists are among the pharmaceuticals used to prevent or alleviate shivering after SA [5].

Despite meperidine’s unique kappa‐receptor action making it a long‐standing antishivering drug, it is linked to opioid‐related AEs such drowsiness, nausea, and respiratory depression [6, 7].

Dexmedetomidine (DEX), a selective *α*2‐adrenergic agonist, has become more popular in the last several years because of its sedative, analgesic, and antishivering effects. When given intravenously before or during SA, it significantly reduces the incidence of shivering [8].

Nefopam, a nonopioid centrally acting analgesic that inhibits serotonin, norepinephrine, and dopamine reuptake, has also demonstrated antishivering efficacy without the respiratory depression or hemodynamic instability commonly associated with opioids or Alpha 2 agonists [9].

Despite growing interest in these agents, there is a notable lack of head‐to‐head comparative studies evaluating the efficacy of intravenous nefopam against both DEX and meperidine in the same surgical population. Most prior studies have either assessed the efficacy of individual drugs or compared only two agents, limiting the ability to draw robust conclusions regarding the optimal pharmacological approach for shivering prevention.

Hence, we postulated that nefopam, which causes less sedation and respiratory depression than DEX and meperidine, is superior in preventing post‐SA shivering. Thus, this research focused on the role of IV nefopam, DEX, and meperidine in averting shivering episodes in adult individuals receiving SA for surgical interventions on the lower abdomen or limbs.

## 2. Patients and Methods

A clinical trial, designed as prospective, randomized, controlled, and double‐blind involved 210 patients of both sexes, aged 21–60 years, with the American Society of Anesthesiologists (ASA) physical status Class I or II, were scheduled for elective lower abdominal or limb surgeries under SA. The study was carried out between October 2024 and April 2025 after obtaining approval from the Ethical Committee of Cairo University Hospital (approval code: N343‐2024) and registration on ClinicalTrials.gov (ID: NCT06627816). Informed consent, documented in writing, was acquired from every participant.

Allergies to any of the study drugs; SA contraindications (such as coagulopathy, systemic infection, increased intracranial pressure, or progressive neurological disorders); thyroid disorders; mental illnesses; severe diabetes; autonomic neuropathies; a history of substance or alcohol abuse; seizure disorders; significant neurological conditions; severe cardiac, pulmonary, renal, or hepatic diseases; and a history of substance abuse were all grounds for exclusion. Further exclusion criteria included obstetric operations, surgeries requiring blood transfusions, and instances with failed or incomplete SA.

Prior to surgical intervention, a comprehensive preoperative assessment, comprising medical history collection, physical examination, and laboratory investigations, was completed for every eligible participant.

### 2.1. Randomization and Blinding

Randomization and blinding procedures were implemented to ensure methodological rigor. Participants were randomly assigned using computer‐generated random numbers (https://www.randomizer.org) and allocated through opaque, sealed envelopes to ensure concealment. The patients were equally distributed into three groups with a 1:1:1 ratio. Group N received IV nefopam at a dose of 0.2 mg/kg (Nopain, Medical Union Pharmaceuticals, Ismailia, Egypt), Group D received IV DEX at a dose of 0.5 μg/kg (Precedex, Hospira Inc., USA), and Group P received IV pethidine (Meperidine, Sunny Pharmaceuticals, Badr City, Cairo, Egypt) at a dose of 0.5 mg/kg.

To maintain blinding, a two‐operator approach was utilized, where one operator prepared and labeled the study drugs, while the other interacted with the patients without knowledge of the group allocation. Data collectors and analysts remained blinded to the group assignments throughout the study. Additionally, patients and their attending caregivers were not informed of the specific study drug they received.

The study medications were prepared as follows: Nefopam 20 mg (1 mL) was diluted with 19 mL of normal saline to yield a 20‐mL solution with a concentration of 1 mg/mL. DEX 50 μg (0.5 mL) was diluted with 19.5 mL of normal saline to yield a 20‐mL solution with a concentration of 2.5 μg/mL. Pethidine 50 mg (1 mL) was diluted with 19 mL of normal saline to yield a 20‐mL solution with a concentration of 2.5 mg/mL.

A temperature probe, electrocardiography (ECG), pulse oximetry, and noninvasive blood pressure monitor were all used to keep tabs on each patient continually throughout the process. Preoperative sedation included administration of 2 mg midazolam following the insertion of an 18‐gauge IV cannula. The operation room’s temperature was carefully controlled by the air conditioning system to remain between 22°C and 24°C. Axillary body temperature was recorded at baseline and then at 30‐min intervals until the conclusion of the observation period.

Each patient received a 10‐mL/kg IV bolus of warmed lactated Ringer solution (37°C) before SA was administered. Subsequently, an intraoperative infusion was started at a rate of 6 mL/kg/h. Using a midline technique with patient sitting, SA was provided at the L3‐L4 or L4‐L5 interspace. A 25‐gauge spinal needle was placed after confirming cerebrospinal fluid flow. An independent anesthesiologist then injected 12–15 mg of 0.5% hyperbaric bupivacaine and 25 μg of fentanyl.

Following the block, patients were positioned supine, and supplemental oxygen was administered via a nasal cannula at a flow rate of 3 L/min until surgery was completed. All IV fluids were kept at 37°C, and patients were covered with a standard surgical blanket. Sensory blockade was evaluated by assessing the loss of cold sensation, ensuring the blockade extended to the T8–T10 dermatomes. For patient administration, the required volume of the respective stock solution—calculated as 0.2 mL per kg of patient body weight, was then withdrawn and diluted into a 100‐mL bag of normal saline (warmed to 37°C). This final solution was infused intravenously over 20 min.

Heart rate, mean arterial pressure, and oxygen saturation were measured every 5 min for 30 min after SA, and thereafter every 15 min.

Shivering was assessed at 10‐min intervals postblock using the scale developed by Wrench et al. [10]. This scale ranges from Grade 0 (no shivering) to Grade 4 (intense, whole‐body shivering).

The time of shivering onset, severity (grade), and duration were recorded. Shivering was continuously monitored, and rescue therapy was administered in cases of Grade 3 or 4 shivering lasting more than 15 min or upon recurrence after initial cessation. Patients then received a single bolus of their originally assigned study drug, identical in dosage to the prophylactic regimen (0.2 mL/kg of the concentrated stock solution diluted in 100‐mL normal saline), given intravenously over 20 min. The response was evaluated for 15 min postinfusion, with shivering reduction to Grade 0 or 1 considered successful. Data collected included the number of rescue doses, total drug volume administered, and time to shivering cessation. The frequency of continuous or recurrent shivering, the response rate (defined as cessation within 15 min), and the total drug dosage used were documented.

At 10‐min intervals, we recorded each patient’s greatest sedation score based on the four‐point scale suggested by Filos et al. [11]. On this scale, Grade 1 is defined as fully awake and attentive, Grade 2 as somewhat sleepy but sensitive to verbal cues, Grade 3 as slightly drowsy but responsive to physical stimuli, and Grade 4 as completely unresponsive. Oversedation was defined as a sedation score of 3 or 4 accompanied by hypoxemia (SpO_2_ ≤ 90%), requiring enhanced monitoring or postoperative intensive care. The incidence of oversedation was also noted.

Other AEs were recorded, including hypotension (defined as MAP below 20% of baseline), bradycardia (HR < 50 beats per minute), hypoxemia (SpO_2_ < 90%), and postoperative nausea and vomiting (PONV). Hypotension was managed using incremental doses of IV ephedrine (10 mg) and fluid boluses of 5 mL/kg. Bradycardia was treated using atropine at 10 μg/kg doses. PONV was managed with 4‐mg ondansetron IV or based on the clinical judgment of the attending anesthesiologist.

Recovery from the block was assessed postoperatively using the pinprick test and motor evaluation of limb movement in the recovery unit.

The primary outcome of the research was the incidence of shivering, defined as a shivering score of 3 or greater, observed throughout the entire study period. Secondary outcomes included various measures such as intraoperative hemodynamic changes, trends in axillary temperature, onset time, severity, duration, and frequency of shivering episodes, as well as recurrence rates. The response to treatment, including the number of boluses of the study drug required, and any AEs were also assessed. Additionally, the highest recorded sedation score and the incidence of oversedation were included as secondary endpoints.

### 2.2. Sample Size Calculation

G.power 3.1.9.2 (Universitat Kiel, Germany) was employed to calculate the sample size. The sample size was calculated according to the incidence of shivering was 36.7% with DEX and was 16.7% with meperidine according to a previous study [12]. Taking into account the 0.05 α error and 90% power of the investigation, and the allocation ratio is 1:1. In order to mitigate dropout, eight cases were added. Consequently, this investigation allocated 210 patients.

### 2.3. Statistical Analysis

SPSS v27 (IBM Corp., Chicago, IL, USA) facilitated statistical computations. Data normality was verified through the Shapiro–Wilk test and visual histogram assessment. Parametrically distributed data were articulated as means ± SD and evaluated using one‐way ANOVA and Tukey’s post hoc analysis. Conversely, nonparametric data were detailed as the median and IQR, with Kruskal–Wallis and Mann–Whitney *U* tests applied for group comparisons. Categorical variables were explored using the Chi‐square test, presented as frequencies and percentages. A *p* value of less than 0.05 (two‐tailed) indicated statistical significance. P1: between N and D groups, P2: between N and P groups, and P3: between D and P groups.

## 3. Results

**Figure 1:**
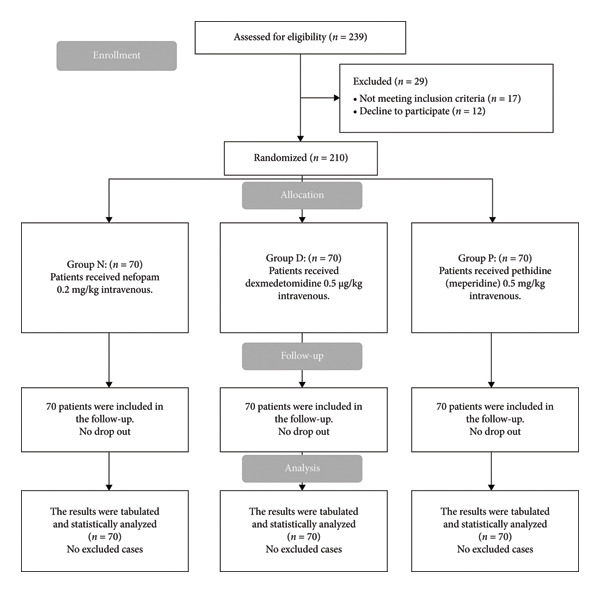
CONSORT flowchart of the enrolled patients.

From the 239 participants initially screened, 17 were excluded for not meeting the inclusion criteria, and 12 opted out of participation. The remaining 210 eligible participants were randomly divided into three groups, each consisting of 70 participants. All enrolled participants (*n* = 210) were randomly assigned into three equal groups of 70 participants each. All enrolled patients completed the study and were included in the statistical analysis (Figure 1).

No notable variation was observed between groups regarding demographic and baseline clinical characteristics (Table 1).

**Table 1:** Demographic data, type, and duration of surgery of the studied groups.

		Group N (*n* = 70)	Group D (*n* = 70)	Group P (*n* = 70)	*P*
Age (years)		42.1 ± 10.41	40.8 ± 10.44	43 ± 7.54	0.386
Sex	**Male**	38 (54.29%)	31 (44.29%)	37 (52.86%)	0.441
	**Female**	32 (45.71%)	39 (55.71%)	33 (47.14%)	
Weight (kg)		80.9 ± 7.9	79.1 ± 9.86	82.1 ± 8.2	0.115
Height (cm)		170.3 ± 6.2	168.7 ± 4.34	169.9 ± 6.26	0.250
Body mass index (kg/m^2^)		28.02 ± 3.64	27.9 ± 3.93	28.6 ± 3.58	0.486
ASA physical status	**I**	43 (61.43%)	37 (52.86%)	39 (55.71%)	0.581
	**II**	27 (38.57%)	33 (47.14%)	31 (44.29%)	
Comorbidities	**Hypertension**	16 (22.86%)	12 (17.14%)	14 (20%)	0.700
	**Diabetes mellitus**	13 (18.57%)	15 (21.43%)	12 (17.14%)	0.806
	**Smoker**	11 (15.71%)	8 (11.43%)	10 (14.29%)	0.756
Type of surgery	**Hemorrhoidectomy**	15 (21.43%)	16 (22.86%)	15 (21.43%)	0.590
	**Hernioplasty**	12 (17.14%)	6 (8.57%)	13 (18.57%)	
	**Cystoscopy and ureteroscopy**	14 (20%)	11 (15.71%)	7 (10%)	
	**Ankle surgeries**	10 (14.29%)	11 (15.71%)	12 (17.14%)	
	**Varicose veins surgeries**	8 (11.43%)	14 (20%)	8 (11.43%)	
	**Knee arthroscopy**	11 (15.71%)	12 (17.14%)	15 (21.43%)	
Duration of surgery (min)		54.1 ± 24.67	59.2 ± 26.2	55.7 ± 26.72	0.496

Note: Data are presented as mean ± SD or frequency (%).

Abbreviation: ASA, American Society of Anesthesiologists.

HR and MAP were comparable at baseline and at 5, 10‐, 15‐, 20‐, and 25‐min post‐SA among all study groups. However, HR and MAP values recorded at 30, 45, 60, 75, 90, and 105 min, as well as at the end of surgery, were significantly increased in Group N and Group P in contrast with Group D (*p* < 0.05), while Groups N and P had similar values. SpO_2_ and core temperature remained statistically similar across all groups at all measurement points (Figure 2).

Figure 2(a) Heart rate, (b) mean arterial pressure, (c) peripheral oxygen saturation, and (d) axillary temperature of the studied groups.

**Figure:**
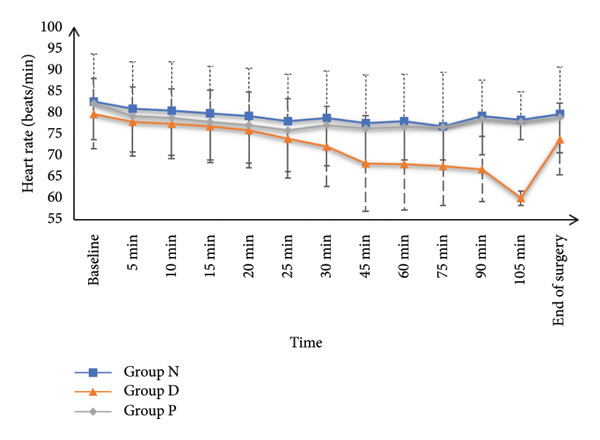
(a)

**Figure:**
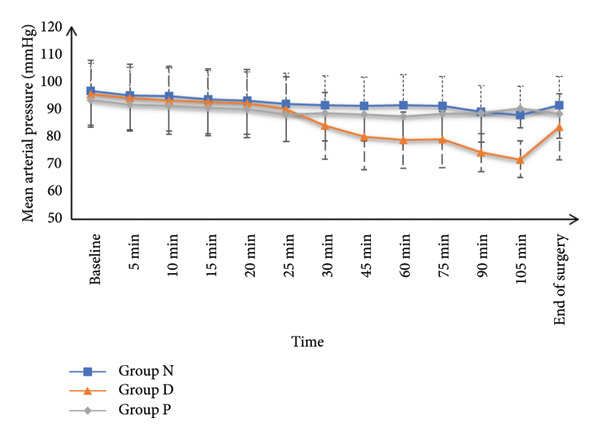
(b)

**Figure:**
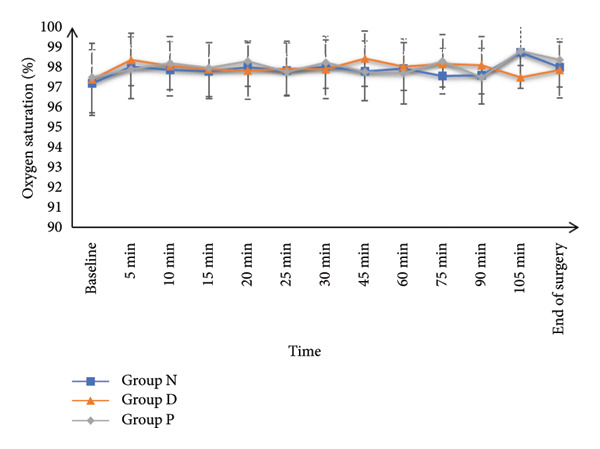
(c)

**Figure:**
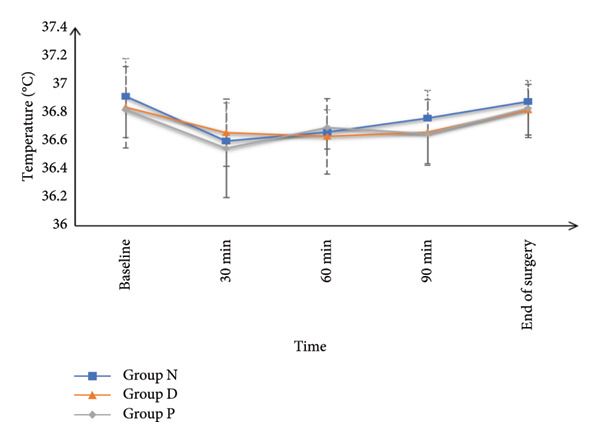
(d)

The three groups had comparable shivering onset, shivering grade, duration of shivering, frequency of rescue drug administration, and total rescue drug dosage. However, the incidence of shivering and the occurrence of continuous shivering episodes were significantly diminished in Group N (*p* < 0.05) in contrast with both Group D and Group P that had comparable incidences (Table 2).

**Table 2:** Shivering characteristics of the studied groups.

	Group N (*n* = 70)	Group D (*n* = 70)	Group P (*n* = 70)	*P*	Post hoc
Incidence of shivering	6 (8.57%)	19 (27.14%)	25 (35.71%)	**< 0.001**	**P1 = 0.004**
					**P2 = 0.001**
					**P3 = **0.275
Onset of shivering (min)	57.5 ± 10.37	48.7 ± 9.98	48.2 ± 7.2	0.167	
Grade of shivering	3 (3–3)	3 (3–4)	4 (3–4)	0.165	
Duration of shivering (min)	11.8 ± 4.88	14.2 ± 5.19	17.2 ± 5.66	0.052	
Continuous shivering	2 (2.86%)	10 (14.29%)	16 (22.86%)	**0.002**	**P1 = 0.016**
					**P2 = 0.004**
					**P3 = **0.192
Frequency of rescue drug	1 (1–1)	1 (1–1)	1 (1–3)	0.08	
Total dose of rescue drug (mL)	20 (20–20)	20 (15–20)	20 (10–40)	0.370	

*Note:* Data are presented as mean ± SD, median (IQR), or frequency (%). Bold values mean that there was a significance when *p* value was < 0.05.

The incidence of hypotension, bradycardia, and sedation was significantly diminished in Groups N and P in contrast with Group D (*p* < 0.05). The incidence of PONV was similar among the groups. Neither hypoxemia nor oversedation was reported in any participant across the study groups. Sedation grades were significantly diminished in Groups N and P in contrast with Group D (*p* < 0.001 and *p* = 0.009, respectively), while comparable sedation grade was detected between Group N and Group P (Table 3).

**Table 3:** Adverse events of the studied groups.

	Group N (*n* = 70)	Group D (*n* = 70)	Group P (*n* = 70)	*p* Value	Post hoc
Hypotension	2 (2.86%)	11 (15.71%)	5 (7.14%)	**0.022**	
Bradycardia	1 (1.43%)	8 (11.43%)	2 (2.86%)	**0.016**	
Postoperative nausea and vomiting	0 (0%)	2 (2.86%)	3 (4.29%)	0.238	
Hypoxemia	0 (0%)	0 (0%)	0 (0%)	—	
Sedation (≥ 3)	0 (0%)	17 (24.29%)	5 (7.14%)	**< 0.001**	
Grade of sedation	1 (1–2)	2 (1–2)	2 (1–2)	**< 0.001**	**P1 < 0.001**
					**P2 = **0.06
					**P3 = 0.009**
Oversedation	0 (0%)	0 (0%)	0 (0%)	**—**	

*Note:* Data are presented as median (IQR) or frequency (%). Bold values mean that there was a significance when *p* value was < 0.05.

## 4. Discussion

The main findings from this trial included that HR and MAP were comparable at baseline and up to 25 min post‐SA; however, from 30 min till the end of surgery, the HR and BP were significantly decreased in Group D in contrast to Groups N and P, with no difference between N and P groups (because the DEX decreases the HR and BP while nefopam and pethidine do not affect the HR and BP significantly). SpO_2_ and core temperature remained similar across all groups. Although shivering onset, grade, duration, and rescue drug use were comparable, the incidence of shivering was significantly diminished in Group N (8.57%) in contrast with Group D (27.14%) and Group P (55.71%). Continuous shivering episodes were also less frequent in Group N. The incidence of hypotension was recorded as 2.86%, 15.71%, and 7.14%, respectively, for Groups N, D, and P, while bradycardia occurred in 1.43%, 11.43%, and 2.86%, respectively, for the same groups. Both AEs were significantly less frequent in Groups N and P in contrast with Group D.

In this research, PONV was comparable among groups. No cases of hypoxemia or oversedation were reported. Sedation scores were significantly diminished in Groups N and P in contrast with Group D, with no difference between Groups N and P.

The rationale for this study is based on evidence that IV DEX (0.5 μg/kg) [13], nefopam (0.15–0.2 mg/kg) [14, 15], and pethidine (0.5 mg/kg) [16] have each demonstrated antishivering efficacy when administered prior to or during SA.

Nefopam has significant antishivering effects, likely due to its multimodal action involving serotonergic, dopaminergic, and adrenergic pathways. Nefopam acts via serotonin and noradrenaline reuptake inhibition, which may enhance thermoregulatory mechanisms by reducing the central drive to shiver. Its nonopioid nature makes it a desirable alternative to opioids, as it is associated with less sedation and respiratory depression [17]. Nefopam reduces shivering by inhibiting serotonin, norepinephrine, and dopamine reuptake and blocking NMDA receptors, altering thermoregulatory monoamine pathways to lower the shivering threshold and gain, thus allowing deeper therapeutic hypothermia without causing sedation or respiratory depression [18] that might explain the diminished incidence of shivering in Group N in contrast with DEX and pethidine in this study.

DEX, an *α*2‐adrenergic agonist, also reduces shivering through its sedative effects, which are mediated by the inhibition of norepinephrine release in the central nervous system. This action reduces the body’s sympathetic response to cold stress, thus preventing shivering. The efficacy of DEX in reducing postanesthetic shivering is well‐documented. For instance, Nazemroaya and Heydari [19] demonstrated in a cesarean section population that intravenous DEX, particularly at a dose of 5 μg/kg, significantly reduced shivering intensity compared to both a lower dose (2.5 μg/kg) and a placebo. However, DEX’s sedative and hypotensive effects are more pronounced in contrast with nefopam [20]. While DEX is effective in shivering prevention, the associated sedation and hypotension may limit its use in clinical practice, especially in patients who require minimal sedative effects or those with preexisting cardiovascular conditions [21].

On the other hand, meperidine (pethidine), a μ‐opioid receptor agonist, also demonstrates efficacy in reducing shivering, but its use is often compromised by the sedative and respiratory depressant side effects typical of opioids [22]. While it is effective in modulating central thermoregulation, the opioid‐related side effects contribute to the increased sedation and hypotension observed in Group P. This is consistent with previous findings, which have emphasized the risk of respiratory depression and sedation with opioids when used for shivering prevention [23].

Our findings regarding the superior antishivering effect of nefopam over DEX appear to contrast with some previous studies that positioned DEX as highly effective. For example, Jabalameli et al. [24], in a study on abdominal surgery patients, reported that DEX (0.5 μg/kg) resulted in a lower incidence of shivering (12.5%) compared to ondansetron, pethidine, and saline, concluding it was associated with less shivering and better hemodynamic stability. This discrepancy with our results, where DEX had a 55.71% shivering incidence, may be attributed to differences in the surgical population (cesarean section and abdominal surgery vs. our mixed lower abdominal/limb surgeries), core temperature management strategies, or the specific metrics of hemodynamic stability assessed. In our study, which implemented aggressive warming with warmed fluids, the inherent advantages of nefopam may have been more clearly demonstrated. Furthermore, our results align with Kasem et al. [25] in highlighting the significant hemodynamic side effects (hypotension and bradycardia) associated with DEX, which were markedly diminished in our nefopam group.

Also, Lv et al. [17] found that administering nefopam as a preventive measure significantly lowered perioperative shivering in patients receiving both general and neuraxial anesthesia.

Additionally, Mohammed’s study [26] found that both DEX and nefopam were effective in reducing shivering, with nefopam showing a significantly faster cessation of shivering (2.35 ± 0.67 min) in contrast with DEX (4.63 ± 1.19 min). The DEX group had increased rates of sedation (24%), hypotension (10%), and bradycardia (14%), while no such effects were observed in the nefopam group. Nefopam exhibited significantly delayed time to first rescue analgesic in contrast with DEX (351.24 ± 19.71 vs. 162.63 ± 9.08 min).

In line with our study, Abdel‐Ghaffar et al. [12] assessed the efficacy, hemodynamic impact, and side effects of DEX (0.5 μg/kg) as opposed to meperidine. They discovered that DEX resulted in a lower rate of complete shivering cessation within 10 min (36.7%) as opposed to meperidine. Additionally, shivering recurred more often in the DEX group (36.7%) than in the one group (16.7%). Nevertheless, DEX was associated with fewer cases of hypotension and bradycardia, and no additional treatments such as ephedrine or atropine were required. Sedation was less common in the DEX group (10%) as opposed to meperidine (13.3%).

Moreover, Kim et al. [27] found that nefopam (0.15 mg/kg) was more effective than meperidine in reducing shivering, causing less sedation (3.1% vs. 15.6%), and maintaining more stable hemodynamics without significant hypotension—unlike meperidine, which reduced MAP below baseline at all intervals—while both drugs had similar rates of nausea, respiratory depression, and oxygen desaturation. The increased dose in our study could have had a more pronounced effect on blood pressure regulation, potentially leading to fewer cases of hypotension in the nefopam group.

The single‐center methodology and limited sample size of this research may limit its generalizability. The exclusion of patients with comorbidities such as cardiovascular, endocrine, or neurological disorders restricts the applicability of the findings to high‐risk populations. The use of subjective assessment tools for evaluating shivering and sedation may have introduced observer bias. The fixed dose of each drug, without titration based on individual response, may not reflect optimal clinical practice. The study focused only on intraoperative and immediate postoperative periods, without assessing delayed AEs or patient‐centered outcomes such as satisfaction or recovery quality.

## 5. Conclusions

IV nefopam demonstrated superior effectiveness as opposed to DEX and pethidine in preventing shivering during SA, significantly reducing both the frequency and duration of shivering episodes. Beyond its effectiveness, nefopam was linked to fewer hemodynamic fluctuations and sedative side effects than DEX. As a nonopioid option, nefopam offers reliable shivering prevention while minimizing common AEs such as bradycardia, hypotension, and sedation, which were more commonly observed with DEX.

## Disclosure

All authors commented on previous versions of the manuscript. All authors read and approved the final manuscript.

## Conflicts of Interest

The authors declare no conflicts of interest.

## Author Contributions

All authors contributed to the study conception and design. Material preparation and data collection and analysis were performed by Emad M. Abdelhafez, Wael El‐Siory, and Dina Turki. The first draft of the manuscript was written by Amany A. Eissa and Emad M. Abdelhafez.

## Funding

The authors received no specific funding for this work.

## Data Availability

The data are available on request from the authors.
